# Zidovudine-induced reversible pure red cell aplasia

**DOI:** 10.4103/0253-7613.66845

**Published:** 2010-06

**Authors:** Anuja Balakrishnan, Rohith Valsalan, Shubha Sheshadri, Vinay R. Pandit, Vikas Medep, Ravindra Kumar Agrawal

**Affiliations:** Departments of Medicine and Pathology, Kasturba Medical College, Manipal University, Manipal, Karnataka, India; 1 Division of Medicine, Lyell McEwin Hospital, Elizabeth Vale, South Australia

**Keywords:** HIV, pure red cell aplasia, Zidovudine

## Abstract

Hematological abnormalities are frequent among human immunodeficiency virus (HIV)-infected patients and may be directly attributable to the virus or may be caused by opportunistic infections, neoplasms or drugs that cause bone marrow suppression or hemolysis. Pure red cell aplasia (PRCA) is an uncommon hematological disorder that causes anemia. We report a 37-year-old male with HIV infection who developed PRCA 6 weeks after commencing Zidovudine and recovered following cessation of the drug. This is the first case of Zidovudine-induced PRCA reported from the Indian subcontinent.

## Introduction

Anemia is well described in acquired immunodeficiency syndrome (AIDS). This has been attributed to the virus or drugs used in the treatment of opportunistic infections. Highly active antiretroviral drugs (HAART) cause suppression of the bone marrow cell precursors, leading to bone marrow failure.[1] One of the rare causes of anemia includes pure red cell aplasia (PRCA), which selectively affects the erythroid bone marrow cells. PRCA has been most commonly associated with thymomas, autoimmune disorders and malignant diseases.[2] Here, we describe a patient who developed PRCA secondary to Zidovudine, which reversed following cessation of the drug. We believe that this is first case of its kind reported from the Indian subcontinent.

## Case Report

We report a 37-year-old male diagnosed with human immunodeficiency virus (HIV) infection who was referred to our center for initiation of HAART. His general and systemic examinations were unremarkable. Baseline investigations revealed hemoglobin 15.2 g, white cell count (WBC) 6,400 cells/mm3, platelet count 135,000 cells/mm3 and mean corpuscular volume 85 fl. Urine examination, renal parameters, liver functions and ultrasound abdomen were within normal limits. His baseline CD4 count and viral load were 112 cells/*μ*l and 750,000 copies/ml, respectively. He was started on Duovir (lamivudine 150 mg + zidovudine 300 mg) twice daily and nevirapine 200 mg once daily on 28/11/2008. Cotrimoxazole was given as primary prophylaxis for Pneumocystis jiroveci infection. Two weeks later, complete blood picture revealed hemoglobin 13.2 g, white cell count 6,400 cells/mm^3^ and platelet count 251,000 cells/mm^3^ with normal liver functions. The dose of Nevirapine was increased to twice daily. During follow-up, 6 weeks after commencing therapy, on 09/01/2009, he presented with exertional breathlessness and fatigue. On examination, he had marked pallor of the oral mucosa and conjunctiva with no evidence of active bleeding from the gastrointestinal, respiratory or genitourinary tracts. There was no icterus or lymphadenopathy. Examination of the cardiovascular system revealed a grade 3/6 ejection systolic murmur. Examinations of the respiratory system and of the abdomen were within normal limits.

**Figure 1:**
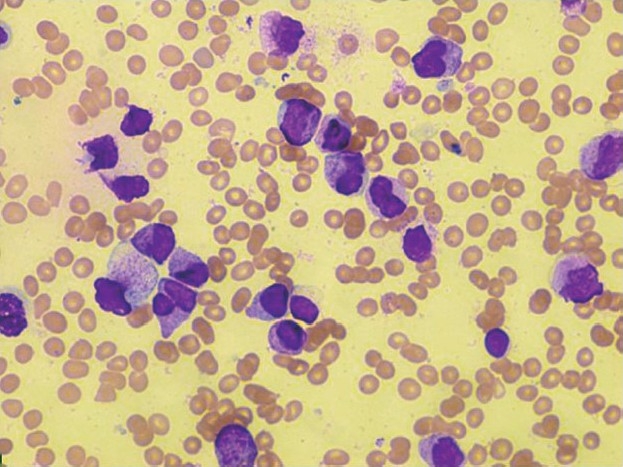
Pure red cell aplasia. Bone marrow aspirate film showing only myeloid precursors (Leishman stain, 40X).

Hematological investigations revealed macrocytic anemia (MCV 103.9 fl) with hemoglobin 7.3 g, reticulocyte count of 0.23% and elevated erythrocyte sedimentation rate (ESR) 40 mm/h. WBC counts and platelet counts were 4,700 cells/mm^3^ and 233,000 cells/mm^3^, respectively. Peripheral smear showed macrocytic anemia with adequate leucocytes and platelets with no evidence of any abnormal cells. Coombs test was negative. Vitamin B_12_ and folate levels were within normal limits.Opportunistic infections and active tuberculosis were excluded. Aerobic and anaerobic cultures of the urine and blood were negative. Venereal Diseases Research laboratory (VDRL). Trepenoma Pallidum Hem agglutination Assay (TPHA) and serology for toxoplasma were negative. Enzyme-linked immunosorbent assay (ELISA) for hepatitis B surface antigen and antibodies to Hepatitis C virus were negative. Parvo virus B19 DNA polymerase chain reaction was negative. Bone marrow trephine biopsy showed hypocellular marrow with markedly suppressed erythropoeisis, with adequate megakaryocytes and leucopoiesis [Figure 1]. Bone marrow stains for acid fast bacilli, fungi and Ebstein Barr virus were negative. There was no evidence of granulomas or giant normoblasts. In spite of three blood transfusions, hemoglobin did not show a marked increase. On 12/1/2009, AZT was stopped. However, lamivudine and cotrimoxazole were continued along with efavirenz and tenofovir. Blood parameters repeated 2 weeks after the new regimen showed hemoglobin 9.4 g, white cell count 4,300 cells/mm^3^ and platelet count 245,000 cells/mm^3^. The reticulocyte count improved to 2.5%. Complete blood picture performed after 6 weeks of therapy showed hemoglobin 12.1 g, white cell count 5,000 cells/cu^3^, platelet count 312,000/mm^3^ and ESR 11 mm/h [Table 1].

**Table 1:** Hematological changes following HAART therapy

*Date*	*Hemoglobin (g)*	*Total count (cells/mm^3^)*	*Reticulocyte count (%)*	*Hematocrit*	*Platelets (cells/mm^3^)*	*MCV (fl)*	*HAART*
28/11/08	15.2	6400			135,000	85	AZT* + 3TC** + NVP***
12/12/08 (2 weeks later)	13.2	6400		38.5	251,000		
09/01/09 (at diagnosis of PRCA)	7.3	4700	0.23	20.9	233,000	103.9	
12/01/09 (3 days later)	5.8	4400		16.8	290,000		AZT stopped TDF**** + EFV***** + 3TC started
26/01/09 (2 weeks of therapy)	9.4	4300	2.5		245,000		
23/02/09 (6 weeks of therapy)	12.1	5000		36.1	312,000		
09/03/09 (8 weeks of therapy)	14.4	6300		42.8	219,000		

AZT* Zidovudine;

3TC** Lamivudine;

NVP*** Nevirapine;

TDF**** Tenofovir;

EFV***** Efavirenz.

## Discussion

PRCA is an uncommon disorder. It is usually associated with autoimmune states, pregnancy, nutritional deficiencies, etc. There are very few case reports of AIDS-associated PRCA in the literature.[3,4]

PRCA is defined when anemia is associated with normal leukocyte and platelet counts, with a corrected reticulocyte count <1% and <5% erythroid precursors in bone marrow in the absence of hemolysis. Our patient fulfilled the above-mentioned criteria. It was possible to exclude other conditions associated with PRCA, including nutritional deficiencies. Parvovirusassociated PRCA, which is supposed to be a frequent cause of anemia in patients with AIDS, was ruled out.[4] There was no evidence of autoimmune hemolysis, which was supported by a negative Coombs test. In our patient, at the time of diagnosis of PRCA, the patient was receiving Zidovudine and Lamivudine. These two drugs have been associated with PRCA.[5–7]

The resolution of PRCA on discontinuation of Zidovudine, as evidenced by an increase in hemoglobin and reticulocyte count, clearly indicates that, in our patient, the erythroid disease was secondary to Zidovudine and not due to Lamivudine.[8,9] Repeat bone marrow aspiration was not performed in the patient. Therefore, we believe that PRCA was secondary to Zidovudine.

PRCA typically occurs within the first 3 months of therapy with Zidovudine or Lamivudine.[5,7] But, there are reports of AZT-induced PRCA occurring even after 4 years of treatment, as described by Weinkove.[10] In this patient, this complication occurred 6 weeks after commencing therapy. There are two mechanisms of PRCA in AIDS, the first being the autoimmune response as a consequence of immune-dysregulated status in AIDS and the second being the myelosuppressive effect of antiretroviral therapy. The possible mechanism for hematosuppression could be due to the synergistic action of Zidovudine and Lamivudine.Most physicians believe that withdrawal of combination of AZT + 3TC is enough as the condition responds to such withdrawal and that bone marrow examination is not mandatory.

Anemia on HAART has been reported in 23% of the Indian patients by Kumaraswamy *et al*.[11] However, there are only a few case reports of Zidovudine-induced PRCA in the literature and, to the best of our knowledge, there are no published reports of Zidovudine-induced PRCA from India. We believe that this is the first reported case of Zidovudine-induced PRCA from the Indian subcontinent, a nation that is placed third in the world in terms of greatest number of people living with HIV.

A survey by UNAIDS in 2007 concluded that there were 2.5 million infected people in the country. It is a fact that Zidovudine is the most frequently used drug in India. Therefore, the chance of HIV-infected patients on HAART suffering from PRCA is high, and this may go unnoticed due to the lack of awareness among the physicians. Therefore, we would like to emphasize that bone marrow aspiration must be performed in all cases of HIV with anemia on HAART to exclude PRCA, as this is the only method to establish diagnosis.

In this patient, Zidovudine-associated PRCA appeared after a short time of exposure to the drug. Considering the fact that Zidovudine is a drug that is widely used in India, we expect more AIDS patients suffering this adverse effect in the near future, but this may go unnoticed if the physicians are not aware of this complication.
